# RNAi-mediated knockdown of Dnmt3a enhances antibody titer in CHO cells up to 200 percent

**DOI:** 10.1186/1753-6561-9-S9-P8

**Published:** 2015-12-14

**Authors:** Anica Schmidt, Anna Wippermann, Raimund Hoffrogge, Thomas Noll

**Affiliations:** 1 Institute of Cell Culture Technology, Bielefeld University, 33615 Bielefeld, Germany

## Background

Increasing cell specific productivity is still an important challenge in today's production of biopharmaceuticals in mammalian cell cultures. During the last two decades various efforts were made to achieve higher yields, e.g. by optimization of process strategies, media development or cell line and metabolic engineering. Nevertheless, some challenges remain for efficient and applicable high titer production processes at industrial scale: the stability of engineered cell lines and the complexity of stable high-titer production processes as well as the characterization for regulatory authorities and strategies for downstream processing [1-3].

We found decreased transcript levels of the *de novo *methyltransferase 3a (Dnmt3a) in independent cultivation processes with increased productivity, i.e. in perfusions under glucose limitation and batch cultivation upon butyrate treatment. To prove the hypothesis that reduced transcript levels of Dnmt3a is responsible for an increase in productivity, we investigated the influence of RNAi-mediated knockdown of Dnmt3a in recombinant CHO cells on cell growth, viability and cellular and process productivity.

## Materials and methods

Cultivations of an IgG1-producing CHO-XL99 clone (provided by Dr. Jeff Hou, AIBN, University of Queensland) and the anti-IL-8 producing CHO DP-12 clone #1934 (ATCC CRL-12445) were performed in customized chemically defined animal-component free media (Xell AG) in perfusion and batch mode. Samples for viable cell densities and viabilities (automated cell counting system CEDEX, Roche Diagnostics) and for determination of IgG1 antibody concentrations (Protein A (Invitrogen) HPLC, using CHO DP-12 standards) were taken daily. Western blots were performed using Dnmt3a rabbit monoclonal antibody (D23G1, Cell Signaling) and AffiniPure goat anti-rabbit IgG (Jackson Immuno Research Laboratories).

To explore the influence of decreased Dnmt3a levels in antibody producing recombinant CHO cell lines, three different siRNAs and one scrambled control were designed (shRNA Sequence Designer (Clontech), siRNA Wizard (Invivogen)). After cloning each siRNA into the pcDNA 3.1 vector (Life Technologies) and transformation of TOP10 *E.coli *cells, the CHO-XL99 cells were transfected using the nucleofector (Nucleofector™ 2b, Lonza) and nucleofector kit V (Lonza). Transfected cells were grown in parallel for 24 h with an untransfected and a scrambled control in customized chemically defined animal-component free media (Xell AG). Samples for analysis of cell growth and viability as well as for monoclonal antibody (IgG1) were taken before and 24 h post-transfection; cell specific productivities were calculated.

## Results

**Figure 1:**
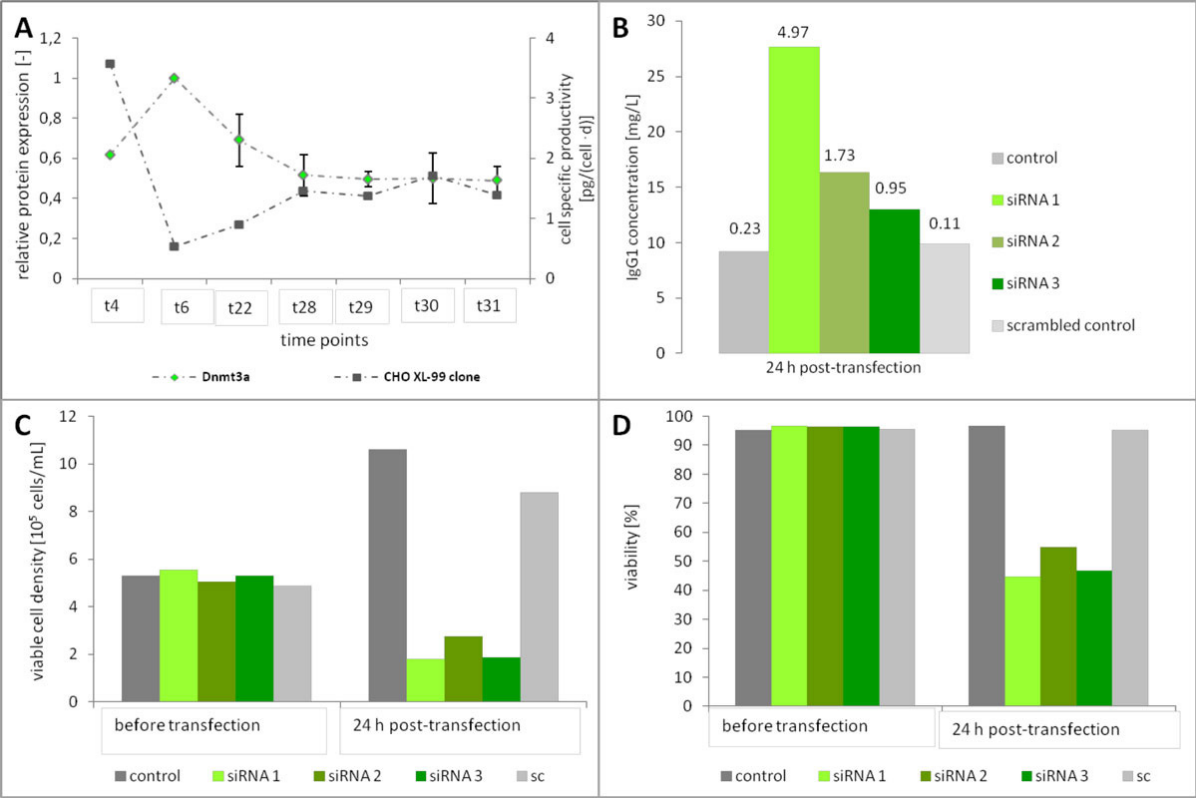
**Correlation between enhanced antibody production and decreased Dnmt3a expression level**. **(a) **Correlation between cell specific productivity of a CHO-XL99 clone in cultivation time course of perfusion and Dnmt3a protein expression level (n = 4, protein expression levels were normalized to t6). **(b) **Average monoclonal antibody concentrations of transient transfected CHO-XL99 cells and untransfected control, n = 2. Numbers above bars represent cell specific productivities [pg/(cell × d)] within 24 h. **(c) **Average viable cell densities of transfected CHO-XL99 cells and untransfected control. **(d) **Average viabilities of transfected CHO-XL99 cells and untransfected control.

After 24 days of cultivation cell specific productivity started to increase in late, glucose-limited phase of CHO-XL99 clone perfusion (data not shown). Corresponding to this, the expression level of Dnmt3a decreased (Figure 1A). Furthermore, upon butyrate treatment cell specific productivity increased in different CHO cell lines and process modi with decreased Dnmt3a expression levels (data not shown).

Based on this, we hypothesized that reduced expression of Dnmt3a is responsible for an increase in productivity under glucose limitation as well as upon butyrate treatment.

RNAi mediated transient silencing of the *de novo *methyltransferase Dnmt3a led to enhanced cell specific productivity of up to 4.95 pg/(cell × d) compared to the control culture (0.23 pg/(cell × d)) and the scrambled control (0.11 pg/(cell × d)) (Figure 1B). Although a strong decrease in cellular viability was observed, product titers were increased by 31-201 % (Figure 1B).

Besides enhanced productivity, transient transfections showed strong negative impact on viable cell densities and viabilities of CHO-XL99 cells. Viable cell densities decreased from 5.3 × 10^5 ^cells/mL to 1.8 × 10^5 ^cells/mL (siRNA 1), 1.9 × 10^5 ^cells/mL (siRNA 2) and 2.8 × 10^5 ^cells/mL (siRNA 2), respectively, 24 h post-transfection (Figure 1C). Viabilities dropped from an initial value of 96 % to 55% (siRNA 2), 49 % (siRNA 3) and 44 % (siRNA 1), respectively (Figure 1 D). The control culture and transfection with scrambled control remained unaffected in all cases (Figure 1B-D).

These results indicated that a reduced expression of Dnmt3a could be responsible for enhanced productivity but also results in a strong decrease in viability and cell density.

## Conclusions

In this study we found that both glucose limitation and butyrate treatment enhance cell specific productivity in CHO DP-12 and CHO-XL99 cells. Moreover, a correlation between enhanced monoclonal antibody production and the expression level of the *de novo *methyltransferase 3a could be observed.

To further explore this phenomenon, we designed several siRNAs and transfected CHO-XL99 cells transiently. This transient knockdown of Dnmt3a enhanced productivity but negatively impacted growth. The increase in monoclonal antibody production might be due to DNA methylation events. To overcome negative side effects on cellular viability and cell growth, stable cell lines using an inducible RNAi system are under preparation. These stable cell lines with inducible knockdown of Dnmt3a will offer the opportunity to have a more detailed view on the mechanisms of enhanced monoclonal antibody production.
